# Molecular Characterization of the *Dwarf53* Gene Homolog in *Dasypyrum Villosum*

**DOI:** 10.3390/plants9020186

**Published:** 2020-02-03

**Authors:** Mikhail Bazhenov, Anastasiya Chernook, Pavel Kroupin, Gennady Karlov, Mikhail Divashuk

**Affiliations:** 1 Laboratory of Applied Genomics and Crop Breeding, All-Russia Research Institute of Agricultural Biotechnology, Timiryazevskaya street, 42, 127550 Moscow, Russia; irbis-sibri@yandex.ru (A.C.); pavelkroupin1985@gmail.com (P.K.); karlovg@gmail.com (G.K.); divashuk@gmail.com (M.D.); 2 Kurchatov Genomics Center of All-Russia Research Institute of Agricultural Biotechnology, Timiryazevskaya str. 42, 127550 Moscow, Russia

**Keywords:** *Dasypyrum*, wheat, distant hybridization, plant height, tillering, strigolactones, sequencing, PCR, marker, introgression

## Abstract

The *Dwarf53 (D53)* gene, first studied in rice, encodes a protein that acts as a repressor of the physiological response of plants to strigolactones—substances that regulate the activity of axillary buds, stem growth, branching of roots and other physiological processes. In this work, we isolated and sequenced the homolog of the *D53* gene in several accessions of the wild grass *Dasypyrum villosum* of different geographical origins, resulting in the discovery of large allelic variety. A molecular marker was also created that allows us to differentiate the *D. villosum D53* gene from common wheat genes. Using this marker and monosomic addition, substitution and translocation wheat lines carrying the known *D. villosum* chromosomes, the *D53* gene was localized on the long arm of the 5V chromosome.

## 1. Introduction

The *D53* gene (*Dwarf53*, previously referred to as *Dwarf-Kyushu-3*, *D-K-3*) was first described in rice due to the short-stemmed dominant mutation resulting from radiation mutagenesis associated with the increased tillering capacity of plants [1]. The gene is localized on the short arm of the 11th chromosome of rice and it has been shown to be non-affecting of either the level of gibberellins or sensitivity to them [2]. Later, it was found that the dominant short-stemmed mutation of the *D53* gene is associated with plant insensitivity to strigolactones, a group of recently discovered phytohormones that inhibit the growth of axillary buds and the protein encoded by this gene plays the role of a repressor of the physiological response of plants to these chemical compounds [3]. The mutant rice DWARF53 protein is more stable in the cell due to disrupted interaction with the E3 ubiquitin-ligase complex, which modifies the protein with the ubiquitin molecule, thereby referring it for degradation by the proteasome [4]. In addition to branching, strigolactones regulate the symbiotic and parasitic relationships of plants, secondary stem growth, leaf aging, seed germination and root development [5].

Plant height, root branching and tilling capacity are important economically valuable properties of wheat, determining its resistance to lodging, the ability to absorb nutrients from the soil and the productivity of individual plants [6,7,8]. Ultimately, all these properties affect crop yields. The study of genes that regulate yield-associated properties is of great practical importance. Many studies show that during the process of wheat domestication and transition from diploid to hexaploid species, allelic gene diversity is greatly narrowed [9]. Therefore, the study of diploid species—the ancestors of modern cultivated wheat and its wild relatives—seems to be the most promising for the purpose of finding new economically valuable alleles.

*Dasypyrum villosum* (L.) Borbás (2n = 14, VV) is a cross-pollinated annual herbaceous plant of the Triticeae tribe, growing in the north-eastern part of the Mediterranean, south-west Asia, Russia and the Caucasus. This is a very viable ruderal plant that is resistant to cold, drought, salinization and also to various wheat diseases including yellow, brown and stem rust, powdery mildew and strawbreaker foot rot. This cereal is a wild relative of wheat. Wheat lines bearing individual chromosomes of *D. villosum,* chromosome arms or fragments thereof were obtained. Many researchers consider *D. villosum* as a valuable source of genes for improving wheat, primarily its resistance to abiotic stress and diseases. It is also known that *D. villosum* has a good tillering capacity and produces a large number of grains with a high protein content [10].

The objective of this research was to study the allelic diversity of the *D53* homolog gene in *D. villosum and* to create molecular genetic tools to further evaluate the effect of the alleles of this gene on economic traits in introgressive common wheat lines. In this work, we sequenced *D53* homolog genes from *D. villosum* accessions of different geographical origins, created a molecular marker that allows us to differentiate the *D. villosum D53* gene from common wheat genes, and, using this marker and a set of monosomic addition wheat lines carrying the definite chromosomes of *D. villosum*, determined the chromosomal localization of this gene.

## 2. Results

**Figure 1:**
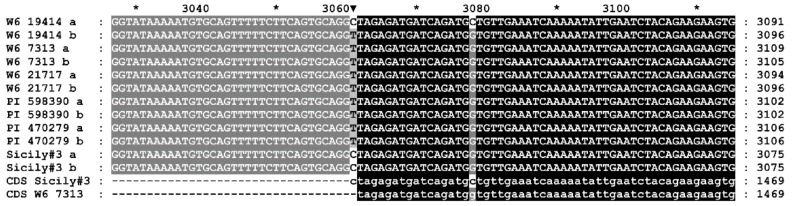
The T/C polymorphism at the putative border of the second intron and third exon of the *D53* gene of *D. villosum.*

The *D53* gene of *D. villosum* was isolated by polymerase chain reaction (PCR) using primers selected on the basis of genomic sequences of common wheat (see Materials and Methods). Sequencing of the PCR products obtained from the six *D. villosum* accessions of diverse geographical origin showed that the *D53* gene is highly polymorphic in this species. Six plants, each taken from individual accession, were found to possess 12 different alleles of this gene (Supplementary File 1). The results of analysis of the next generation sequencing data clearly show that the genes of all the studied sample plants were heterozygous. However, it is impossible to determine the polymorphic nucleotide variants in each allele of a heterozygous plant accurately if the polymorphic loci of the gene are at a distance greater than the sequencing read length. The differences between alleles are represented by both insertions or deletions localized in introns and numerous single nucleotide variants scattered throughout the gene. In particular, a single nucleotide T/C polymorphism was detected at the putative border of the second intron and third exon, which presumably affects mRNA splicing. The ‘T’ variant was found to be present in most plants and the ‘C’ variant was present in Sicyly#3 and W6 19414 plants in the heterozygous state (Figure 1). 

**Figure 2:**
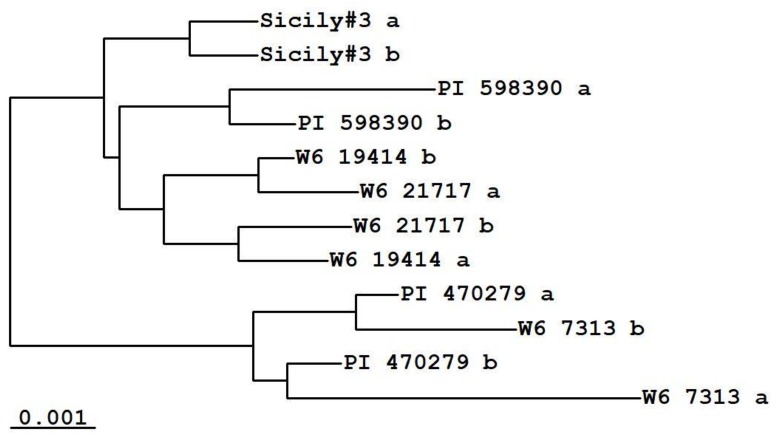
The phylogram based on the first intron of the *D53* gene in various accessions of *D. villosum*. Distances are calculated using the Tajima and Nei model [11]. The letters *a* and *b* denote two alleles of each plant.

A phylogenetic analysis based on the nucleotide sequence of the first intron of the gene reflects the geographical origin of the *D. villosum* accessions. Accessions from Turkey and Greece were grouped into a separate branch; within the other branch, Bulgarian and Crimean accessions were very similar, differing from the accessions from Sicily and the former USSR (Figure 2). The phylogram shows that, in some cases, the alleles of different accessions of *D. villosum* are more similar to each other than the alleles of one accession. This may indicate cross-pollination between natural populations of this species or cross-pollination between accessions that occurred during their propagation under artificial conditions. Additionally, to some extent, this could be due to an artifact of the gene sequence assembly algorithm from the short reads. The results reflect the extremely high genetic diversity and the allogamy of *D. villosum.*

In total, 109 single-nucleotide polymorphisms, referring to 11 different alleles, were found in the protein-coding sequence of the gene (Supplementary File 2). Two sister alleles of the Sicilian accession of *D. villosum* (Sicyly#3), provided by A.J. Lukaszewski, were found to be identical in terms of protein-coding sequence. A total of 47 of the 109 polymorphisms led to predictable changes in the amino acid sequence. Analysis of amino acid substitutions using the PROVEAN online service showed that three of them are significant for the functioning of the protein (score less than –2.5). These are substitutions of D717A, S742F and E848V, caused by the single-nucleotide variants A2150C, C2225T and A2543T, respectively (coordinates are indicated in the protein-coding sequence). However, these three mutations were rare and were found only in one of the accessions, W6 19414, PI 470279 or PI 598390, respectively, in a heterozygous state. Other amino acid substitutions were characterized as neutral.

The BLAST-search for sequences homologous to *D. villosum D53* in the nucleotide database of the National Center for Biotechnology Information (NCBI) showed that it is most similar to the homologs of common wheat. A phylogenetic analysis carried out according to the Tajima and Nei [11] model showed the same distances between the consensus sequence of *D. villosum D53* and all three homologs of this gene in common wheat. At the same time, we found some differences that allow us to differentiate the genes of wheat and *Dasypyrum*. The differences between the *D53* wheat genes and their homologs in *D. villosum,* that are specific for botanical species, are various small mutations (nucleotide substitutions, insertions or deletions), affecting more frequently one and rarely two or three, neighboring nucleotides. Based on one of the dinucleotide mutations, we selected PCR primers to detect the presence of the *D53* gene of *D. villosum* in the background of the wheat genome (Figure S1). 

**Figure 3:**
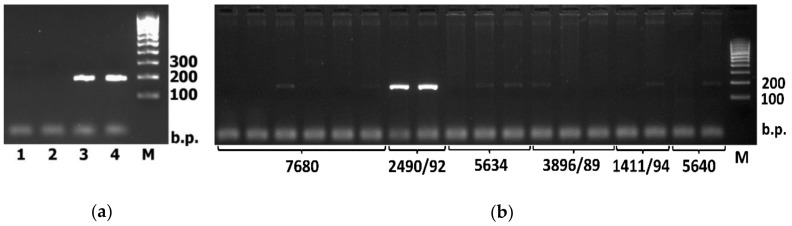
The polymerase chain reaction (PCR) marker for detection of the *D. villosum* D53 gene in the wheat background: (**a**) Electrophoresis of the PCR products obtained with primers DvD53-F and DvD53-R. Tracks 1, 2, using DNA of Brigada (1) and Vassa (2) common wheat varieties; 3, 4, using *D. villousm* DNA of W6 21717 (3) and PI 598390 (4). M is a DNA size standard M-100 (Sintol LLC); (**b**) An example of electrophoresis of PCR products obtained using the DNA of soft wheat lines carrying individual *D. villosum* chromosomes or its fragments. Line 2490/92 has a 5V(5D) chromosome substitution.

Testing of the created PCR marker on the DNA of wheat varieties and *D. villosum* accessions confirmed its performance (Figure 3a). The developed marker was used to establish the *D. villosum* chromosome, which carries the *D53* gene. To do this, PCR was performed using the above-mentioned primers and DNA of monosomic addition, substitution and translocation lines of Chinese Spring common wheat bearing certain *D. villosum* chromosomes or their fragments (Table S1, Figure 3b).

Intensive PCR amplification of the marker fragment, comparable to that obtained with *D. villosum* DNA, was in the 7681, 2333/89 and 2490/92 wheat lines carrying the 5V chromosome. PCR using DNA of the 5638 line with a 5DL.5VS translocation (carrying a short arm of the 5V chromosome) gave a negative result. It follows that the *D53* gene of *D. villosum* is located on the long arm of the 5V chromosome. This result is consistent with the chromosome localization of the *Dwarf53* gene in wheat. A BLAST search among scaffolds related to specific arms of wheat chromosomes [12] showed that scaffolds related to 5BL, 5DL and 5AL chromosome arms have the greatest homology to the *D53* gene sequence.

## 3. Discussion

*D. villosum* is a wild cereal that is able to grow in harsh soil and climatic conditions, which is resistant to drought, salinization, cold and a number of economically significant diseases of cultivated cereals [13]. The distant hybridization of *Triticum dicoccoies* (Körn. ex Asch. and Graebn.) Schweinf. and *D. villosum* allowed amphidiploids to be obtained that cross well with soft wheat [14]. Thus, it became possible to use the genetic material of this wild species to improve one of the most globally significant crops. The main method of introducing valuable genes from *D. villosum* into the wheat genome is to create disomic-addition, chromosome-substitution and, as the most valuable option, translocation lines of wheat that carry individual fragments of *D. villosum* chromosomes. *Pm21* and *PmV* powdery mildew resistance genes introduced from *D. villosum* into common wheat as parts of T6V#2S·6AL and T6V#4S·6DL translocations have already been successfully used in the development of many commercial varieties [15]. Additionally, *Dasypyrum* was used in wheat breeding as a source of wheat streak mosaic virus resistance genes [16], *Pm62* powdery mildew resistance genes as part of the T2BS.2VL#5 translocation [17], the *Pm55* gene [18] and the *Sr52* stem rust resistance gene, which is effective against the Ug99 race [19]. The alien genetic material of *Dasypyrum* can also contribute to improving the yield and quality of wheat grains [20,21].

The *D. villosum D53* gene selected by us for this study is involved in the regulation of the tillering of cereals and, probably, branching of the root system [5]. Tillering capacity is one of the main components of wheat productivity [7]. *D. villosum* is known to have greater tillering capacity than wheat [13]. Branching of the roots contributes to better absorption of water and nutrients from the soil under conditions of their deficiency [8,22]. At the same time, the *D53* gene also has some effect on the length of the stems [2]. Thus, the transfer of this gene from *D. villosum* to wheat can hypothetically give an improvement in wheat according to the above economic characteristics.

Due to the quite high similarity of the nucleotide sequence, we managed to isolate the *D53* gene of *D. villosum* using primers selected on the basis of genomic sequences of common wheat. As a result of sequencing, we showed that the *D53* gene has a large allelic diversity in *D. villosum*. All plants whose genes were sequenced in this study were heterozygous. Twelve alleles of the studied gene were found, 11 of which had a unique protein-coding sequence. These results indicate that *D. villosum* has an incomparably richer variety of allelic variants of genes than modern wheat varieties and this diversity has not yet been fully studied. At the same time, we should focus not only on the diversity of alleles between different geographically distant populations of a given species but also on the study of a rather large intrapopulation diversity.

The differences between the *D53* homologs of *D. villosum* and wheat are mainly presented by single nucleotide substitutions and substitutions affecting two to three neighboring nucleotides and to a lesser extent, by the presence of small insertions and deletions. Based on one of these differences, we created a molecular marker that allows us to detect the presence of the *D. villosum D53* gene in the wheat genome environment. This marker helped us establish that the *D53* gene in *D. villosum* is located on the long arm of the 5V chromosome. A previous study showed that the 5V chromosome of *D. villosum* improves drought tolerance by stimulating the development of a deeper and branched root system [22]. However, that study used the 5638 line, bearing the short arm of the 5V chromosome and, according to our results, not carrying the *D53* gene. The effect of the long arm of the 5V chromosome on the root system has not been studied yet.

This research creates prerequisites for further study of the economic value of the *D. villosum D53* alleles that could be introduced into common wheat. The developed marker will make it possible to track the introgression of this gene into the wheat genome and study its effect on various features and properties of plants.

## 4. Materials and Methods 

*D. villosum* accessions of different geographical origin were used for sequencing the homolog of the *D53* gene (Table 1).

The plant DNA was extracted from the leaves using the cetyltrimethylammonium bromide-based protocol [23]. The DNA of only a single plant from each accession of *D. villosum* was used for PCR amplification and sequencing of the *D53* gene.

The sequences of the *D53* homoeologous genes of common wheat (TraesCS5A02G155000, TraesCS5B02G153200, TraesCS5D02G159900) were retrieved from the genome assembly IWGSC RefSeq v1.0 using BLAST [24] based on the gene sequence accession KY363316 (GenBank database, NCBI). The primers needed for amplification of the *D53* gene of *D. villosum* were designed using the Primer BLAST [25] to match the conserved regions among the three wheat homologs and the PCR product size, so as not to exceed 1500 base pairs. During primer design, the melting temperatures of the oligonucleotides were calculated according to the SantaLucia model [26] (Table 2). The PCR performed using these primers and the DNA of the *D. villosum* gave amplicons of a size close to the expected for wheat. Together, the PCR products covered the whole sequence of the *D53* gene.

The PCR mixture composition was as follows (final concentrations of the reagents are given): 1x buffer for LR-Plus polymerase (pH = 9.3), 1.5 mM MgCl_2_, 0.2 mM of each dNTP, 2 µM of each primer, 0.04 U/µL LR-Plus polymerase (Sileks), 0.02 U/µL Taq-Plus polymerase (Sileks) and 4 ng/µL of matrix DNA. The volume of the PCR mixture was 25 µL. The temperature conditions were as follows: 94 °C for 5 min.; 36 cycles of 94 °C for 30 s, 58 °C for 30 s, 72 °C for 2 min and 72 °C for 5 min.

The quality and quantity of the obtained PCR products was analyzed by electrophoresis in 1.5% agarose gel, with the addition of ethidium bromide. The gels were visualized in ultraviolet light and documented using the Gel Doc XR+ system (BioRad). 

The PCR products obtained using the DNA of each single *D. villosum* plant were mixed together in equal volumes in a separate tube and submitted for sequencing. Sequencing performed on the Illumina MiSeq system was ordered in “OOO Genomed.” The DNA libraries were prepared using Swift 2S™ Turbo DNA Library Kits. In the process of library preparation, the content of each tube was tagged with an individual barcode. The resulting reads were sorted according to barcodes, thus keeping the data for each plant separate.

The quality of the reads was assessed using FastQC software [27]. For each *D. villosum* plant, the contigs were assembled separately from paired-end reads using the SPAdes 3.13.0 program package [28]. The contigs were aligned to the reference sequence of the *TaD53-5D (TraesCS5D02G159900)* gene of wheat using the ‘contig editor’ module of GeneStudio 2.2 [29]. The resulting alignment was transformed from ‘pretty view’ to ‘fasta’ format using an original Microsoft Windows application [30]. The alignment was imported to GeneDoc 2.7 [31], where after discarding the reference sequence and a contig sequence generated by GeneStudio, a consensus sequence of the *D. villosum D53* gene was obtained. The consensus (raw) sequence could have contained some small inaccuracies. To correct them, the initial reads were mapped on a raw sequence using SNAP [32]. To detect inaccuracies, the Freebayes program [33] was used and detected alternative variants were applied to the sequence using the Bcftools package [34]. Being allogamous, *D. villosum* plants are supposed to be heterozygous for most genes. To reveal the ‘alternative allele’ sequence in each plant, the last obtained sequence was used as a reference for one more procedure of mapping the reads, polymorphism calling and alternative variants incorporation. Thus, two sequences of the *D53* gene for each *D. villosum* plant (accession) were generated. The resulting sequences were placed in the NCBI GenBank database under the accession numbers MN862624–MN862635.

The generated sequences of D53 gene were aligned to each other using the Clustal W 1.83 algorithm in the alignment editor of GeneStudio. The phylogenetic analysis, using the model of Tajima and Nei [11], was performed using the PHYLYP 3.698 program package [35].

Prediction of the splicing patterns was performed using the Augustus online version, indicating an organism like *Triticum aestivum* [36]. Translation of the coding DNA sequence to the protein amino-acid sequence was done in GeneDoc 2.7. Prediction of the functional significance of the amino-acid changes was done using the PROVEAN online service [37].

To detect the presence of the *D. villosum D53* gene in the wheat genome background we developed the PCR primers DvDw53-2F: 5’-TGCACGCACAGCATGTTAAATA-3’ and DvDw53-2R: 5’-ACCTGTGTTGATCCTCTGGGA-3’. The PCR was conducted under the following conditions: 94 °C for 5 min, 36 cycles of 94 °C for 30 s, 60 °C for 30 s, 72 °C for 30 s and 72 °C for 5 min. The composition of the reaction mix was as follows: 1x *Taq*-buffer, 2.5 mM MgCl_2_, 0.2 mM of each dNTP, 2 µM of each primer, 0.05 U/µL of *Taq*-polymerase and 4 ng/µL of matrix DNA. The PCR products were detected by electrophoresis in 1.5% agarose gel with TBE buffer stained with ethidium bromide and visualized in ultraviolet light.

The developed PCR marker was tested on the DNA of *D. villosum* accessions and the common wheat varieties Brigada and Vassa and applied on a series of monosomic addition, substitution and translocation lines of the Chinese Spring wheat variety, carrying the known chromosomes of *D. villosum* or their fragments. The wheat lines carrying alien genetic material used in our study are listed in Table S1.

## Figures and Tables

**Table 1:** *Dasypyrum villosum* accessions used in this study.

Accession	Place of Collection	Source
W6 19414	Bulgaria	W6
W6 7313	Greece	W6
W6 21717	Crimea	W6
PI 598390	former USSR	W6
PI 470279	Turkey	W6
Sicily#3	Italy, Sicily	AJL

W6: Western Regional Plant Introduction Station, Washington State University. The accessions were provided through the U.S. National Plant Germplasm System. AJL: the accession provided by A. J. Lukaszewski.

**Table 2:** The PCR-primers used for amplification of the fragments of the *Dasypyrum villosum D53* gene.

Primer Pairs, Sequence Direction is 5’ -> 3’	Tm	Expected Amplicon Size for Wheat Subgenomes		
		A	B	D
D53-F1: CGTGGTTTATAAGCAAGCAATCCA D53-R1: GGGGAACTTGGACAGGAAGG	60	1239	1281	1263
D53-F2: TACCTCACCTTCCTGTCCAAGTT D53-R2: TCTTACCCTTTTCATCAAGCTGT	58	1619	1459	1248
D53-F3: ACTTACTGCATCTGGGTTGATAA D53-R3: ATAGCTTCACACCTTGATTGCAT	58	810	1013	811
D53-F4: CGGTGTCAACAGTGCAATGAT D53-R4: GCCTCCTGAAGCTGGTGAAT	60	959	958	959
D53-F5: ATTCACCAGCTTCAGGAGGC D53-R5: ACTACCGTGGACTAGCTACC	60	1403	1397	1398

Tm: melting temperature of oligonucleotides calculated according to the SantaLucia model [26].

## Supplementary Materials

The following are available online at https://www.mdpi.com/2223-7747/9/2/186/s1, Supplementary File 1: The alignment of the *D53* genes of common wheat and *D. villosum* in FASTA format, Supplementary File 2: The alignment of the CDS part of the D53 genes of common wheat and *D. villosum* in FASTA format, Figure S1: A part of the alignment of the *D53* genes of common wheat and *D. villosum*. The PCR primers DvD53-F and DvD53-R selected for the regions differing between wheat and *D. villosum* are highlighted, Table S1: The Chinese Spring wheat lines carrying alien genetic material from *D. villosum* used for mapping of the *D53* gene.
