# Supplementary material for: Molecular Characterization of the Dwarf53 Gene Homolog in Dasypyrum Villosum
Source: Plants (Basel). 2020 Feb 3;9(2):186. doi: 10.3390/plants9020186 (PMC7076371; doi:10.3390/plants9020186)
Supplement: Supplementary file 1 [file plants-09-00186-s001.zip › Figure S1.pdf]

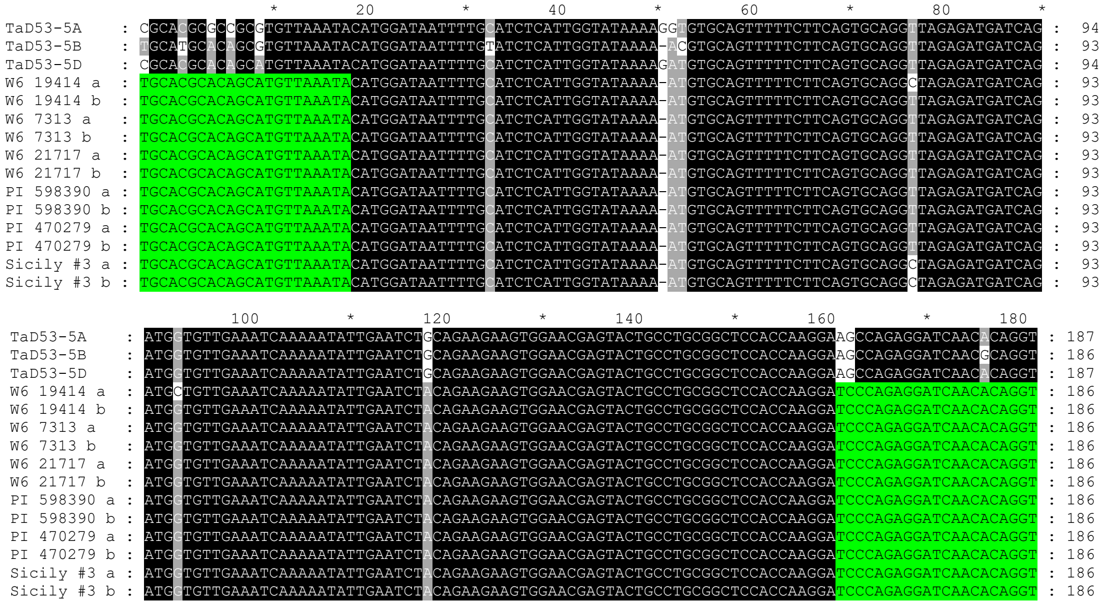

Figure S1. A part of the alignment of the *D53* genes of common wheat (*TaD53-5A*, *TaD53-5B, TaD53-5D*) and *D. villosum* (accession numbers are indicated). The PCR primers DvD53-F and DvD53-R selected for the regions of the *D53* gene differing between wheat and *D. villosum* are highlighted in green.
