# Supplementary material for: Molecular Characterization of the Dwarf53 Gene Homolog in Dasypyrum Villosum
Source: Plants (Basel). 2020 Feb 3;9(2):186. doi: 10.3390/plants9020186 (PMC7076371; doi:10.3390/plants9020186)
Supplement: Supplementary file 1 [file plants-09-00186-s001.zip › Table S1.pdf]

Table S1. The Chinese Spring wheat lines carrying alien genetic material from *D. villosum* used for mapping of the *D53* gene.

Line	Rearrangement type[1]	Chromosome	Source[2]	Marker amplification[3]
7677	A	1V#3	KSU	-
7679	A	3V#3	KSU	-
7680	A	4V#3	KSU	-
7681	A	5V#3	KSU	++
7682	A	6V#3	KSU	-
7683	A	7V#3	KSU	-
7510	A	2V#1	KSU	-
7511	A	4V#1	KSU	-
7513	A	6V#1	KSU	-
7514	A	7V#1	KSU	-
3891/89	S	1V(1A)	AJL	-
3896/89	S	1V(1D)	AJL	-
86/11	S	3V(3B)	AJL	-
1360/07	S	3V(3D)	AJL	-
2333/89	S	5V(5D)	AJL	++
2490/92	S	5V(5D)	AJL	++
1411/94	S	6V(6B)	AJL	-
1415/94	S	6V(6A)	AJL	-
3889/89	S	7V(7A)	AJL	-
6661	S	6V#2(6A)	KSU	-
5585	T	T6AL·6V#2S	KSU	-
5594	T	T4DS·4V#3L	KSU	-
5615	T	T1DS·1V#3L	KSU	-
5616	T	T1DL·1V#3S	KSU	-
5634	T	T2BS·2V#3L	KSU	-
5636	T	T3DL·3V#3S	KSU	-
5637	T	T3DS·3V#3L	KSU	-
5638	T	T5DL·5V#3S	KSU	-
5639	T	T7DL·7V#3S	KSU	-
5640	T	T7DS·7V#3L	KSU	-
1438/94	T	6BS·6VL	AJL	-
3214/96	T	6AS·6VL	AJL	-
853/11	T	3V·3BL+3B	AJL	-

[1] Rearrangement type: A – addition, S – substitution, T – translocation. [2] Source: KSU - Kansas State University; AJL - A. J. Lukaszewski, personal communication. [3] Marker amplification: "++" – strong amplification, "-" – weak or no amplification.
